# Exposure to ambient fine particulate matter is associated with changes in fasting glucose and lipid profiles: a nationwide cohort study

**DOI:** 10.1186/s12889-020-08503-0

**Published:** 2020-04-03

**Authors:** Woo-young Shin, Jung-ha Kim, Gyeongsil Lee, Seulggie Choi, Seong Rae Kim, Yun-Chul Hong, Sang Min Park

**Affiliations:** 1 Department of Family Medicine, Chung-ang University Medical Center, Seoul, 06973 Republic of Korea; 2 Department of Family Medicine and Biomedical Sciences, College of Medicine, Seoul National University, 101 Daehak-ro, Jongno-gu, Seoul, 03080 Republic of Korea; 3 Department of Biomedical Sciences, College of Medicine, Seoul National University, Seoul, 03087 Republic of Korea; 4 Department of Medicine, College of Medicine, Seoul National University, Seoul, 03087 Republic of Korea; 5 Department of Preventive Medicine, College of Medicine, Seoul National University, Seoul, 03087 Republic of Korea; 6 Institute of Environmental Medicine, Seoul National University Medical Research Center, Seoul, 03087 Republic of Korea; 7 Environmental Health Center, College of Medicine, Seoul National University, Seoul, 03087 Republic of Korea

**Keywords:** Air pollution, Particulate matter, Glucose, Lipid, Cohort study

## Abstract

**Background:**

Ambient fine particulate matter is a rising concern for global public health. It was recently suggested that exposure to fine particulate matter may contribute to the development of diabetes and dyslipidaemia. This study aims to examine the potential associations of ambient particulate matter exposure with changes in fasting glucose and lipid profiles in Koreans.

**Method:**

We used the data from the National Health Insurance Service–National Sample Cohort (NHIS-NSC), a nationwide database representative of the Korean population. A total of 85,869 individuals aged ≥20 years were included. Multiple regression analyses were conducted to assess the associations between exposure to particulate matter and changes in fasting glucose and lipid profiles at 2-year intervals after adjusting for confounders.

**Results:**

Significant associations were observed between an increase in interquartile range for particulate matter < 2.5 μm in diameter (PM_2.5_) and elevated levels of fasting glucose and low-density lipoprotein cholesterol (*p* for trend = 0.015 and 0.010, respectively), while no association for particulate matter sized 2.5–10 μm in diameter (PM_10–2.5_) was noted after adjusting for the other covariates. Sub-group analyses showed stronger associations in individuals who were older (≥60 years) or physically inactive.

**Conclusions:**

Fine particulate matter exposure affects worsening fasting glucose and low-density lipoprotein cholesterol levels, with no evidence of an association for coarse particulate matter.

## Précis

Fine particulate matter exposure affects to worsen fasting glucose and low-density lipoprotein cholesterol levels, with stronger associations in individuals who were older or physically inactive.

## Background

Cardiovascular disease and diabetes have become the major causes of mortality and public health concerns around the world. It is estimated that approximately 20 million people die of these diseases annually [1]. Similarly, these diseases accounted for approximately 24.3% of the causes of death among the Korean population in 2016, and the rates of morbidity and mortality related to these diseases are continuously increasing [2, 3]. Accordingly, there have been various studies and approaches to identify or manage them in Korea, as well as worldwide.

Several countries, including South Korea, that experienced industrialization and urbanization in recent decades have aggravated atmospheric quality and air pollution. These conditions have raised concern regarding the leading risk factors for the global burden of diseases [4, 5]. Ambient particulate matter (PM) air pollution accounted for approximately 7.5% of deaths and was the sixth highest factor contributing to disability-adjusted life years in 2016 [1]. PM refers to a widespread air pollutant consisting of a mixture of variable particles, including sulfates, nitrates, organic materials, particle-bound water, metals, or even biological components suspended in the air [6]. Its ambient exposure is ubiquitous and involuntary, affecting large populations. Several studies on the health effects of PM air pollution have been actively conducted in many countries. PM exposure is well known for its detrimental effects on the cardiovascular and respiratory systems and total mortality [5, 7, 8]. Some epidemiological studies have recently suggested that exposure to ambient PM may contribute to harmful health effects on metabolic systems, such as increased risks of hyperglycaemia and dyslipidaemia [9, 10], which are known as well-established cardiometabolic risks with strong evidence for developing diabetes and cardiovascular events [11].

However, limited studies have focused on the relationship of PM exposure with hyperglycaemia and dyslipidaemia, with most being conducted as cross-sectional designs for short-term periods among relatively few participants. Furthermore, to the best of our knowledge, few studies have explored the effects of PM concentrations on individual changes in cardiometabolic risks using a large cohort. Therefore, the aim of this study was to examine the associations between the concentrations of PM exposure and changes in levels of blood glucose and lipid profiles among Korean adults using a nationwide cohort.

## Methods

### Data source and study population

The National Health Insurance Service–National Sample Cohort (NHIS-NSC) is a nationwide population-based cohort with a substantial volume of information regarding citizens’ health examinations and utilization of health insurance among a representative Korean population [12]. The NHIS-NSC database was constructed for research and policy development by the National Health Insurance Service (NHIS). This study is based on data from the NHIS-NSC obtained from 2002 to 2013, which includes participants’ sociodemographic variables and health examination results (Project number: NHIS-2018-2-135).

A total of 1,006,481 individuals were randomly selected using a systematic stratified sampling method, maintaining approximately 2.2% of the entire eligible Korean population since 2002, followed-up until 2013 [12]. Of these, 223,491 participants aged < 20 years old were excluded in this study, considering factors influencing children or adolescents’ exposure to PM. Children or adolescents’ exposure to environmental contaminants, including PM, is expected to be different from adults due to differences in their physiologic characteristics and behavioural patterns [13]. Therefore, this study was designed and conducted to evaluate the health effects of PM exposure in adults. In addition, we excluded 527,082 participants whose data on PM_10_ or PM_2.5_ levels were not available during the study periods, as this study aimed to provide potential evidence of the size-specific effects of PM exposure by evaluating the adverse effects of both fine and coarse PM on changes in laboratory values. Of the remaining 255,908 participants, we excluded 169,125 participants who did not take two biennial health examinations consecutively during 2010–2013. A total of 85,869 (43,595 men and 42,274 women) individuals were finally analysed after excluding participants who had missing data on the results of health examinations and questionnaires.

Informed consent was obtained for the NHIS-NSC from all participants. This study was approved by the institutional review board of Seoul National University (IRB number: E-1803-045-928).

### Assessment of exposure to particulate matter

We obtained atmospheric monitoring data for the daily mean concentrations of hourly measured PM with aerodynamic diameters of ≤10 μm (PM_10_) and ≤ 2.5 μm (PM_2.5_) from the National Ambient Air Monitoring Information System in the Ministry of Environment of Korea [14]. The hourly exposure concentrations were measured using the beta-ray absorption method, following the standard reference protocol from the Korean Air Pollutants Emission Service [15]. Based on participants’ administrative residential codes, we identified and matched the nearest monitoring site to each residence at the district level. The 2-year average concentrations of each district-specific PM_10_ and PM_2.5_ exposure for the period of 2010 to 2011, corresponding to 2 years before the follow-up health examination visits between 2012 and 2013, were calculated using their daily mean values at a total of 49 national monitoring sites placed in the residential areas. Daily mean levels of coarse particulate matter (PM_10–2.5_) were calculated by subtracting the daily mean levels of PM_2.5_ from those of PM_10_. For the analysis stratified by quantitative exposure to each PM_10–2.5_ and PM_2.5_, their 2-year mean concentrations were classified into four levels using quartiles.

### Main outcomes and other variables

Information on participants’ sociodemographic characteristics (age, sex, residential area, and income level) and the results of self-reported health questionnaires, physical examinations, and biochemical tests were gathered from the first and second biennial health examinations during the study period.

The main outcomes for this study were changes in the levels of fasting blood glucose (FBG) and lipid profiles 2 years after PM exposure, calculated by subtracting each laboratory value obtained during the first health examination (baseline) from those of the second health examination (follow-up). These blood samples were taken after fasting for at least 12 h.

Data on systolic and diastolic blood pressure, anthropometric indices (height and weight), and health behaviours (smoking status, alcohol consumption, and physical activity) as well as sociodemographic information were collected from the first health examination records as covariates and baseline characteristics of the study population. Body mass index (BMI) was calculated by dividing weight in kilograms by the square of height in metres and categorized as underweight (< 18.5 kg/m^2^), normal (18.5 to 22.9 kg/m^2^), overweight (23.0 to 24.9 kg/m^2^), or obese (≥25.0 kg/m^2^) according to the Asia Pacific criteria of the World Health Organization [16]. Based on the responses of the self-reported questionnaire, we classified smoking status into ‘never’, ‘ex-’, and ‘current’ smokers. Drinking habits were categorized by the frequency of alcohol consumption per week. The physically ‘active’ group was defined as those who engaged in moderate activities for ≥30 min in a day, at least 5 days a week, or in vigorous activities for ≥20 min in a day, at least 3 days a week [17]. The physically ‘inactive’ group was defined as not meeting these criteria. The status of comorbid diseases for the past 10 years was assessed at baseline with the Charlson comorbidity index (CCI) developed by Quan et al., which has been used broadly in public health research [18].

### Statistical analysis

All analyses were conducted for each estimate of PM_10–2.5_ and PM_2.5_ concentrations separately, and the participants were categorized into four groups by quartiles of the mean PM levels for the 2 years of the first health examination period. To compare between-group characteristics of the population, categorical variables were expressed as percentages of the population using Pearson chi-squared tests, while continuous variables were expressed as the means with their standard deviations using analysis of variance (ANOVA). Multiple linear regression analyses were conducted to estimate associations of exposure to PM with changes in FBG and lipid profiles at a 2-year interval after adjusting for potential confounding covariates, including age, sex, smoking status, alcohol consumption, physical activity, BMI, income level, and CCI. The associations were expressed as the adjusted mean differences of the change in laboratory outcome values with 95% confidence interval (95% CI) based on quartiles of PM exposure levels. We also performed stratified analyses based on the following characteristics of the participants at baseline: CCI score (0 versus ≥1); FBG levels (< 126 mg/dL versus ≥126 mg/dL); LDL-C levels (< 160 mg/dL versus ≥160 mg/dL); physical activity (active versus inactive) and age (< 60 years versus ≥60 years) as potential effect modifiers, by examining the interactions of the potential effect modifiers and PM_2.5_ exposure separately. To test the stability of the associations, we further conducted sensitivity analyses by excluding participants whose FBG, LDL-C, and blood pressure were equal to or higher than the normal range (FBG ≥ 126 mg/dL, LDL-C ≥ 160 mg/dL, and blood pressure ≥ 140/90 mmHg) in the heath examination at baseline. Additional sensitivity analyses were performed after excluding participants who had different residences from baseline over the study period. Statistical data mining and analyses were conducted using SAS version 9.4 (SAS Institute Inc., Cary, NC, USA) and STATA version 15.1 (Stata Corp., TX, USA), and *P* values of < 0.05 were considered statistically significant.

## Results

The mean concentrations of exposure to coarse PM (PM_10–2.5_) and fine PM (PM_2.5_) at baseline were 22.81 ± 4.64 μg/m^3^ and 25.94 ± 3.56 μg/m^3^, respectively. Of the 85,869 participants, 50.77% were men, and 60.37% were aged 40–64 years. At baseline, each group, according to quartiles of PM_10–2.5_ and PM_2.5_, had similar proportions of participants regarding smoking, alcohol consumption, and physical activity status. The sociodemographic and clinical characteristics of the participants according to quartiles of each PM_10–2.5_ and PM_2.5_ at baseline are presented in Table 1.

**Table 1:** Baseline characteristics of study population according to quartiles of each PM_10–2.5_ and PM_2.5_ levels at individual’s residential districts

	Whole NHIS-NSC	All study population	Quartiles of PM_10–2.5_ levels				*P* value	Quartiles of PM_2.5_ levels				*P* value
			Q1	Q2	Q3	Q4		Q1	Q2	Q3	Q4	
Mean level of PM_10–2.5_, μg/m^3^ ± SD		22.81 ±4.64	17.59 ±3.67	21.92 ±0.66	24.34 ±0.83	28.14 ±3.17	< 0.001	22.12 ±5.49	23.24 ±2.68	23.85 ±3.87	21.86 ±5.73	< 0.001
Mean level of PM_2.5_, μg/m^3^ ± SD		25.94 ± 3.56	25.88 ±4.18	25.23 ±1.97	26.30 ±2.83	26.43 ±4.47	< 0.001	21.78 ± 1.83	24.97 ± 0.62	26.89 ± 0.70	30.98 ± 2.15	< 0.001
Population, *n*	1,006,481	85,869	23,354	22,292	19,114	21,109		22,717	21,622	22,959	18,571	
Men, %	50.02	50.77	50.66	50.79	51.03	50.64	0.858	50.25	50.9	50.45	51.65	0.026
Age, %							< 0.001					< 0.001
0–19 years	22.21	–										
20–39 years	29.26	25.05	21.97	26.51	26.32	25.76		22.76	28.01	26.2	22.97	
40–64 years	36.87	60.37	62.71	60.08	58.90	59.39		62.2	57.24	59.14	63.28	
≥65 years	11.66	14.59	15.32	13.40	14.78	14.85		15.04	14.75	14.66	13.75	
Income level, %							< 0.001					< 0.001
Lowest	31.85	27.40	28.42	26.61	27.33	27.19		27.57	25.91	26.6	29.93	
Lower middle	28.57	28.77	28.44	28.58	28.70	29.41		28.34	27.37	29.01	30.64	
Higher middle	25.18	28.25	28.77	28.18	27.37	28.53		28.94	27.5	28.62	27.83	
Highest	14.41	15.57	14.37	16.62	16.61	14.87		15.15	19.23	15.78	11.6	
Smoking status, %							0.164					< 0.001
Never smoker	62.10	62.82	62.37	63.00	62.67	63.28		62.86	63.22	63.47	61.52	
Ex-smoker	14.52	15.64	15.63	15.88	15.46	15.55		15.83	16.14	15.57	14.89	
Current smoker	23.38	21.54	22.00	21.13	21.87	21.17		21.31	20.63	20.96	23.6	
Alcohol consumption, per week, %							< 0.001					< 0.001
None	53.03	51.93	53.39	50.89	50.90	52.35		52.3	49.81	51.62	54.33	
1–2 times	33.20	34.79	33.71	35.68	35.63	34.30		34.37	36.28	35.01	33.33	
3–4 times	9.74	9.62	9.18	9.88	9.73	9.71		9.67	10.17	9.84	8.63	
≥5 times	4.02	3.66	3.72	3.55	3.74	3.64		3.66	3.74	3.54	3.71	
Physical activity^*a*^, %							0.055					0.053
Active	30.00	20.53	21.00	20.34	19.98	20.70		21.16	20.33	20.24	20.34	
Inactive	70.00	79.47	79.00	79.66	80.02	79.30		78.84	79.67	79.76	79.66	
BMI^*b*^, kg/m^2^, mean ± SD	23.74 ± 3.26	23.64 ± 3.15	23.64 ±3.12	23.62 ±3.17	23.70 ±3.16	23.60 ±3.15	0.011	23.69 ± 3.15	23.62 ± 3.19	23.65 ± 3.16	23.58 ± 3.08	0.002
Underweight, %	3.86	3.71	3.37	3.92	3.69	3.89	0.001	3.35	4.17	3.79	3.52	< 0.001
Normal, %	38.99	39.98	40.52	39.84	39.31	40.15		39.81	39.7	39.77	40.78	
Overweight, %	24.60	25.33	25.38	25.53	24.91	25.43		25.29	25.02	25.19	25.9	
Obese, %	32.55	30.98	30.74	30.71	32.09	30.53		31.54	31.11	31.25	29.8	
BP, mmHg, mean ± SD												
Systolic BP	122.50 ± 15.15	121.76 ± 14.90	122.23 ±14.93	121.51 ±14.95	122.03 ±15.11	121.29 ±14.61	< 0.001	121.60 ± 14.66	121.48 ± 15.03	121.69 ± 15.00	122.38 ± 14.91	0.001
Diastolic BP	76.19 ± 10.11	75.56 ± 10.08	75.71 ±9.96	75.40 ±10.16	75.64 ±10.29	75.51 ±9.93	0.005	75.52 ± 9.95	75.45 ± 10.30	75.57 ± 10.11	75.73 ± 9.94	0.045
FBG, mg/dL, mean ± SD	97.94 ± 24.08	97.17 ± 22.82	97.24 ±23.34	96.86 ±21.93	97.12 ±22.92	97.48 ±23.08	0.039	97.63 ± 22.55	96.57 ± 22.20	97.42 ± 22.63	97.02 ± 24.06	< 0.001
TC, mg/dL, mean ± SD	195.15 ± 37.05	195.76 ± 36.41	195.95 ±36.70	195.41 ±36.05	195.22 ±36.18	196.41 ±36.64	0.003	196.45 ± 36.62	195.28 ± 35.99	196.17 ± 36.25	194.97 ± 36.79	< 0.001
TG, mg/dL, mean ± SD	132.83 ± 92.89	128.58 ± 89.37	129.89 ±90.65	127.75 ±89.27	130.62 ±89.85	126.15 ±87.52	< 0.001	128.03 ± 90.00	127.77 ± 87.65	127.58 ± 88.62	131.43 ± 91.43	< 0.001
HDL-C, mg/dL, mean ± SD	55.84 ± 24.43	55.76 ± 15.36	55.73 ±15.39	55.83 ±16.40	55.83 ±15.88	55.68 ±13.62	0.687	55.80 ± 15.80	55.69 ± 15.56	55.69 ± 15.60	55.90 ± 14.25	0.435
LDL-C, mg/dL, mean ± SD	114.06 ± 36.04	114.71 ± 34.33	114.76 ±34.65	114.45 ±33.59	113.82 ±35.04	115.74 ±34.07	< 0.001	115.57 ± 34.72	114.28 ± 33.45	115.55 ± 34.71	113.13 ± 34.32	< 0.001
CCI, %							< 0.001					< 0.001
0	33.87	28.83	27.09	29.08	30.29	29.17		27.71	30.96	28.64	27.97	
1	38.19	31.92	31.66	31.89	31.76	32.39		31.23	31.69	32.02	32.92	
≥2	27.94	39.25	41.25	39.03	37.95	38.43		41.07	37.35	39.34	39.11	

Note: PM_10–2.5_, particulate matter sized 2.5-10 μm in aerodynamic diameter; PM_2.5_, particulate matter with aerodynamic diameter ≤ 2.5 μm; *NHIS-NSC* National Health Insurance Service–National Sample Cohort, *n*, number, *SD* standard deviation, *BMI* body mass index, *BP* blood pressure, *FBG* fasting blood glucose, *TC* total cholesterol, *TG* triglycerides, *HDL-C* high-density lipoprotein cholesterol, *LDL-C* low-density lipoprotein cholesterol, *CCI* Charlson comorbidity index

^*a*^ Participants were divided into active or inactive group based on the total minutes of each level of physical activities. Active group was specified as performing for ≥30 min in a day at least 5 days a week in moderate activities, or for ≥20 min in a day at least 3 days a week in vigorous activities

^*b*^ Participants were categorized as underweight, normal, overweight, and obese by Asia pacific criteria of World Health Organization

We found no significant correlations of the changes in FBG and all the lipid profiles, including total cholesterol (TC), triglycerides (TG), high-density lipoprotein cholesterol (HDL-C), and low-density lipoprotein cholesterol (LDL-C), with increases in PM_10–2.5_ concentrations at 2-year intervals after adjusting for other covariates (*P* for trend≥0.05 for each of FBG, TC, TG, HDL-C, and LDL-C). When assessing the associations of PM_2.5_ exposure and the changes in laboratory values, we observed a positive correlation of elevations of both FBG and LDL-C levels (*P* for trend = 0.015 and 0.010, respectively) after adjusting for other covariates. However, there was no significant association of an increase in exposure to PM_2.5_ with changes in TC, TG, and HDL-C levels (*P* for trend≥0.05 for TC, TG, and HDL-C). The associations of PM_10–2.5_ and PM_2.5_ exposure with changes in FBG and lipid profiles are shown in Table 2.

**Table 2:** Association between each PM_10–2.5_ and PM_2.5_ levels and changes in individual’s concentration of fasting glucose and lipid profiles

	Quartiles of PM_10–2.5_ levels				*P* for trends
	Q1	Q2	Q3	Q4	
Mean level, μg/m^3^ ± SD	17.59 ± 3.67	21.92 ± 0.66	24.34 ± 0.83	28.14 ± 3.17	
Mean difference of individual’s concentration, mg/dL (95% CI)					
FBG	0.956	0.588	1.214	0.598	0.429
	(0.693–1.218)	(0.318–0.857)	(0.923–1.505)	(0.322–0.875)	
TC	0.446	0.175	0.316	0.408	0.998
	(0.027–0.864)	(−0.254–0.605)	(− 0.147–0.779)	(− 0.032–0.848)	
TG	− 0.504	− 0.322	−0.743	0.023	0.620
	(−1.518–0.510)	(− 1.363–0.718)	(−1.865–0.380)	(− 1.044–1.090)	
HDL-C	− 0.034	−0.116	− 0.350	−0.146	0.214
	(−0.228–0.161)	(− 0.315–0.083)	(−0.565--0.135)	(− 0.350–0.059)	
LDL-C	0.669	0.361	0.587	0.907	0.360
	(0.248–1.090)	(−0.071–0.793)	(0.122–1.052)	(0.464–1.351)	
	Quartiles of PM_2.5_ levels				*P* for trends
	Q1	Q2	Q3	Q4	
Mean level, μg/m^3^ ± SD	21.78 ± 1.83	24.97 ± 0.62	26.89 ± 0.70	30.98 ± 2.15	
Mean difference of individual’s concentration, mg/dL (95% CI)					
FBG	0.550	0.975	0.687	1.180*	0.015
	(0.283–0.816)	(0.702–1.249)	(0.422–0.953)	(0.886–1.475)	
TC	0.192	0.46	0.076	0.696	0.312
	(−0.232–0.616)	(0.024–0.896)	(−0.347–0.498)	(0.227–1.166)	
TG	−0.404	−0.524	− 0.235	−0.365	0.862
	(−1.432–0.625)	(−1.581–0.533)	(−1.259–0.790)	(− 1.505–0.774)	
HDL-C	− 0.219	0.026	− 0.208	− 0.212	0.690
	(−0.416--0.022)	(− 0.176–0.229)	(− 0.404--0.012)	(−0.430–0.006)	
LDL-C	0.341	0.618	0.316	1.384*	0.010
	(− 0.086–0.768)	(0.180–1.056)	(− 0.109–0.741)	(0.910–1.857)	

Note: Models were adjusted for age, sex, income level, smoking status, alcohol consumption status, *BMI* physical activity, and *CCI* score. *PM*_*10–2.5*_, particulate matter sized 2.5-10 μm in aerodynamic diameter, *PM*_*2.5*_ particulate matter with aerodynamic diameter ≤ 2.5 μm; n, number, *SD* standard deviation, *CI* confidential interval, *FBG* fasting blood glucose, *TC* total cholesterol, *TG* triglycerides, *HDL-C* high-density lipoprotein cholesterol, *LDL-C* low-density lipoprotein cholesterol

* *P* value < 0.05 of multiple regression for quartile 4 group, compared to quartile 1 group as a reference

The results of stratified analyses are presented in Table 3. Significant modified effects were observed for the physical activity status and age. We found that significant elevations in FBG and LDL-C levels were associated with increments of PM_2.5_ exposure in all subgroups, except the groups with FBG ≥126 mg/dL, LDL-C ≥ 160 mg/dL, or physically active groups at baseline. In association with PM_2.5_ exposure for age-specific groups, significant changes in FBG and LDL-C levels were found only among groups aged ≥60 years (*P* for trend = 0.014 and 0.004, respectively). In the sensitivity analyses, no substantial change was observed from the main analysis, and the associations of PM_2.5_ exposure with increases in FBG and LDL-C levels remained positive and significant among participants whose FBG, LDL-C, and blood pressure at baseline were within the normal range (*P* for trend< 0.001 for FBG and 0.028 for LDL-C, respectively). Restricting the analyses to participants who had the same addresses over the study period also showed the significant associations of PM_2.5_ exposure with increases in FBG and LDL-C levels (*P* for trend = 0.006 for FBG and 0.019 for LDL-C, respectively).

**Table 3:** Stratified analyses of association between PM_2.5_ concentrations and change in levels of fasting glucose and lipid profiles

	Mean difference of individual’s concentration, mg/dL (95% CI)	Quartiles of PM_2.5_ levels				*P* for trends	*P*_inter_
		Q1	Q2	Q3	Q4		
CCI							0.207
0	Mean level, μg/m^3^ ± SD	21.839 ± 1.790	24.888 ± 0.574	26.609 ± 0.513	30.563 ± 2.221		
	FBG	0.892 (0.491–1.293)	0.940 (0.536–1.345)	1.082 (0.675–1.490)	1.928 (1.520–2.337)	< 0.001	
	LDL-C	1.437 (0.703–2.171)	1.924 (1.185–2.664)	2.077 (1.332–2.823)	2.934 (2.183–3.684)	0.006	
≥1	Mean level, μg/m^3^ ± SD	21.779 ± 1.834	24.972 ± 0.621	26.890 ± 0.701	30.979 ± 2.148		
	FBG	0.550 (0.283–0.816)	0.975 (0.702–1.249)	0.687 (0.422–0.953)	1.180 (0.886–1.475)	0.015	
	LDL-C	0.341 (−0.086–0.768)	0.618 (0.180–1.056)	0.316 (− 0.109–0.741)	1.384 (0.910–1.857)	0.010	
FBG at baseline, mg/dL							0.152
< 126	Mean level, μg/m^3^ ± SD	21.779 ± 1.832	24.972 ± 0.619	26.887 ± 0.699	30.970 ± 2.139		
	FBG	1.711 (1.506–1.915)	2.120 (1.910–2.329)	1.877 (1.673–2.080)	2.636 (2.409–2.862)	< 0.001	
	LDL-C	0.825 (0.395–1.255)	0.947 (0.507–1.388)	0.689 (0.261–1.117)	1.767 (1.289–2.244)	0.022	
≥126	Mean level, μg/m^3^ ± SD	21.776 ± 1.866	24.972 ± 0.650	26.932 ± 0.729	31.107 ± 2.290		
	FBG	−18.051 (−20.907--15.196)	−17.233 (−20.279--14.187)	−17.943 (−20.826--15.061)	−21.123 (−24.260--17.986)	0.176	
	LDL-C	−7.618 (−9.899--5.337)	−4.763 (−7.186--2.340)	−5.667 (−7.985--3.350)	−4.444 (−6.956--1.931)	0.105	
LDL-C at baseline, mg/dL							0.052
< 160	Mean level, μg/m^3^ ± SD	21.786 ± 1.832	24.970 ± 0.621	26.885 ± 0.699	30.964 ± 2.137		
	FBG	0.592 (0.314–0.871)	1.075 (0.791–1.360)	0.759 (0.482–1.036)	1.237 (0.930–1.544)	0.018	
	LDL-C	3.519 (3.127–3.910)	3.721 (3.321–4.120)	3.540 (3.150–3.930)	4.605 (4.172–5.037)	0.002	
≥160	Mean level, μg/m^3^ ± SD	21.731 ± 1.845	24.993 ± 0.617	26.930 ± 0.715	31.157 ± 2.279		
	FBG	0.295 (−0.602–1.192)	0.034 (− 0.934–1.002)	0.068 (− 0.830–0.967)	0.342 (− 0.694–1.378)	0.986	
	LDL-C	−30.200 (− 32.319--28.082)	−31.971 (− 34.252--29.690)	− 30.564 (− 32.685--28.443)	− 32.279 (− 34.729--29.829)	0.366	
Physical activity^*a*^							0.032
Active	Mean level, μg/m^3^ ± SD	21.726 ± 1.848	24.883 ± 0.578	26.842 ± 0.733	31.094 ± 2.263		
	FBG	0.612 (0.006–1.218)	1.354 (0.691–2.017)	0.613 (0.019–1.207)	1.092 (0.409–1.775)	0.629	
	LDL-C	0.422 (−0.490–1.334)	0.748 (− 0.246–1.742)	0.380 (− 0.513–1.274)	1.425 (0.397–2.454)	0.271	
Inactive	Mean level, μg/m^3^ ± SD	21.794 ± 1.830	24.973 ± 0.621	26.884 ± 0.621	30.949 ± 2.117		
	FBG	0.534 (0.238–0.830)	0.854 (0.550–1.157)	0 .738 (0.444–1.032)	1.210 (0.883–1.536)	0.009	
	LDL-C	0.322 (−0.161–0.805)	0.577 (0.083–1.070)	0.328 (− 0.151–0.807)	1.353 (0.819–1.887)	0.021	
Age, years							0.004
< 60	Mean level, μg/m^3^ ± SD	21.795 ± 1.819	24.969 ± 0.612	26.859 ± 0.686	30.953 ± 2.133		
	FBG	0.846 (0.530–1.163)	1.143 (0.823–1.464)	1.049 (0.736–1.362)	1.130 (0.786–1.475)	0.301	
	LDL-C	1.932 (1.413–2.452)	2.085 (1.560–2.610)	1.606 (1.092–2.120)	2.512 (1.945–3.078)	0.377	
≥60	Mean level, μg/m^3^ ± SD	21.754 ± 1.858	24.977 ± 0.637	26.941 ± 0.723	31.024 ± 2.176		
	FBG	0.049 (−0.426–0.524)	0.707 (0.207–1.208)	0.096 (−0.383–0.574)	1.259 (0.717–1.802)	0.014	
	LDL-C	−2.344 (−3.081--1.607)	−1.810 (−2.587--1.033)	−1.824 (− 2.568--1.080)	−0.530 (− 1.374–0.315)	0.004	

Note: Models were adjusted for age, sex, income level, smoking status, alcohol consumption status, *BMI* physical activity, and *CCI* score. *PM*_*2.5*_, particulate matter with aerodynamic diameter ≤ 2.5 μm; *n*, number, *SD* standard deviation, *BMI* body mass index, *BP* blood pressure, *FBG* fasting blood glucose, *LDL-C* low-density lipoprotein cholesterol, *CCI* Charlson comorbidity index, *P*_inter_, *P* value for the interaction terms

^*a*^ Participants were divided into active or inactive group based on the total minutes of each level of physical activities. Active group was specified as performing for ≥30 min in a day at least 5 days a week in moderate activities, or for ≥20 min in a day at least 3 days a week in vigorous activities

## Discussion

In this study, we evaluated the associations of exposure to ambient PM_10–2.5_ and PM_2.5_ with changes in the levels of fasting glucose and lipid profiles at 2-year intervals in a representative Korean population using a nationwide cohort. There was a significant relationship of higher concentrations of ambient PM_2.5_ based on an individual’s residential district with elevations in FBG and LDL-C levels after 2 years of exposure among the study population. However, no association of PM_10–2.5_ levels, as coarse particles, with changes in FBG and lipid profiles was found in this study. To our knowledge, this is the first study to provide direct evidence of the size-specific effects of PM in Asia by evaluating the adverse metabolic effects of both fine and coarse PM on changes in laboratory values.

Several studies provide strong evidence that shows that increases in ambient PM levels lead to increased incidence rates of metabolic diseases, which include but are not limited to diabetes and dyslipidaemia [19–21]. Furthermore, another study by Yitshak Sade et al. suggested that increases in the levels of FBG, glycosylated haemoglobin, and LDL-C are associated with increase in PM_10_ and PM_2.5_ concentrations at 3-month intervals from exposure [22]. Similarly, this study provides strong evidence for elevated FBG and LDL-C levels when exposed to PM_2.5_ at 2-year intervals with large-scale demographics. The well-established mechanism of PM- associated metabolic changes in glucose or lipids has not been clarified. Possible explanations for the observed metabolic adverse effects of PM might include systemic inflammation related to activated pro-inflammatory cytokines and subsequent oxidative stress, as well as endothelial dysfunction. A previous epidemiological study of children showed a higher BMI among those who lived in areas with high levels of air pollution, including PM exposure [23]. It was suggested that exposure to PM may affect the deposition of visceral adipose tissue [24, 25]. The impaired lipid metabolism seems to be related to oxidative stress and systemic inflammation through pro-inflammatory cytokine release induced by the accumulation and impairment of visceral adipose tissue by PM exposure [10, 26]. Sun et al. suggested endothelial dysfunction as another explanatory mechanism of these PM-associated cardiometabolic changes, which was found in animal models [27]. It may also help explain the development of cardiovascular morbidity, as well as cardiometabolic risks in association with PM exposure [28]. For changes in TC and TG levels, no significant relationship with PM exposure was obtained after adjusting for covariates in this study. Previous studies have suggested that higher concentrations of ambient PM were correlated with lower concentrations and impaired functions of HDL-C, as well as poor health outcomes of TC and TG concentrations [22, 29–31]. However, their long-term effects could not be identified clearly and were inconsistent regarding the relationship between PM and lipid profiles, as most were conducted during short-term periods. More studies are needed to identify the exposure periods that show metabolic effects and their mechanism in the future.

We found no significant relationship between PM_10–2.5_, which comprises more coarse components than PM_2.5_, and changes in FBG and lipid profile levels. This finding suggests that PM may contribute to the development of diabetes and dyslipidaemia at a disproportional intensity between PM_10–2.5_ and PM_2.5_. It was well documented that long-term exposure to PM_2.5_ exerted more remarkable and negative health effects on morbidity and mortality to some organ systems in humans compared with PM_10_ or PM_10–2.5_ exposure [32–36]. However, few studies on the size-specific metabolic effects of PM have been conducted, particularly in Asia. PM_2.5_ consists of smaller particles that can more easily penetrate and move into the lungs and vessels and has more pollutants emitted from anthropogenic sources such as traffic, industrial processes, or combustion of fuels than those of PM_10–2.5_ [33]. It induces more systemic and harmful health effects on the body with a larger surface and more toxicity compared with PM_10–2.5_. Similarly, our study obtained different results by diameter of PM, indicating a significant association of individual elevation in FBG and lipid levels with higher levels of PM_2.5_ in 2 years after exposure. In previous studies, it was suggested that PM_2.5_ exposure levels were correlated with changes in insulin sensitivity and systemic inflammation [37, 38]. PM_2.5_ may have more evident adverse effects on systemic metabolic regulation than PM_10–2.5_, by inducing impairment of pancreatic β-cell function or aggravation of insulin resistance and metabolic dysregulation, particularly at 2-year intervals from exposure.

We observed significant modifying effects of physical activity and age on the associations of ambient PM_2.5_ exposure with changes in FBG and LDL-C levels. It was showed weaker associations of PM with elevations in FBG and LDL-C levels among the physically active group. It is well known that physical inactivity increases the risk of many non-communicable diseases including cardiovascular diseases and type 2 diabetes with strong evidence, potentially leading to stiffening of the arteries [39, 40]. Regular physical exercise reduces overall cardiometabolic risk, although some physiological reactions to exercise over a few hours or days may transiently cause a greater degree of PM deposition within the lungs by rapid and deep inhalation [41, 42]. Therefore, our findings highlight the potential protective effects of regular physical activity against the development of diabetes and dyslipidaemia in association with exposure to fine PM. Regular exercise appears to help attenuate the harmful cardiometabolic effects in association with ambient PM air pollution, according to the data. We also found that the associations of ambient PM_2.5_ exposure with changes in FBG and LDL-C levels were more pronounced among individuals aged ≥60 years in this study. This finding validates that the elderly population may be more vulnerable to the harmful effects of air pollution, particularly PM_2.5_ exposure, given the evidence that susceptible groups with chronic diseases or the elderly had a higher risk of air pollution-related detrimental health effects than others [36, 43, 44].

Our study indicated discrepancies in the significance of associations between fine PM and alterations in FBG and lipid profiles according to their baseline laboratory values among sub-groups. It was found that the group with a normal range of FBG or LDL-C had a stronger correlation with concentrations of PM_2.5_ exposure than the groups with higher concentrations of FBG or LDL-C. This may be explained by the latent poor metabolic conditions with uncollected medication histories as potential confounding factors in the groups with abnormal laboratory values. The results from sub-groups according to CCI scores were consistent and support the sensitivity of the findings in this study.

Although the study remains reliable, a few limitations should be considered. Assessments of exposure to air pollutants were conducted using data from residential area-specific monitoring sites, which were the nearest stations from individual addresses available at the district level, although the area specific monitoring sites are reliable because the characteristics of the Korean Peninsula near areas may have varying PM data. In addition, we identified and matched the nearest monitoring site to each residence based on baseline records. Thus, the analyses were conducted under the assumption that the proportion of participants who had different addresses from baseline over the study period was negligible. It is recommended that in future studies, long-term exposure should be measured with fine-scale address data, considering changes in participants’ residences over the study period. We calculated and used the levels of PM_10–2.5_ by subtracting PM_2.5_ from PM_10_ to compare the effects of fine PM with those of coarse particles due to the unavailability of measured data on PM_10–2.5_ using monitoring systems [45]. This estimation may induce a larger random error in measurements, but it would be unlikely to affect the statistical power for evaluating the PM_10–25_ effects with exposure estimates of individuals on a large scale of samples. Although we adjusted for several potential confounders, the possibility of other confounders from unmeasured variables cannot be completely ruled out, and the observed associations were likely to have been affected in part by residual confounding factors. Furthermore, the information on medication use was uncollectable due to privacy reasons, and it might have resulted in under- or over-estimation. These confounders need to be addressed to verify these results in further studies. Due to the characteristics of self-reported values in a few of our data, there may be possible response bias. We cannot conclude a direct relationship between ambient PM exposure and laboratory changes, as our study included an observational retrospective design. Thus, we cannot determine whether PM exposure actually affects changes in laboratory values. However, this concern might be mitigated by our large sample size, increasing the statistical power. Health effects of PM exposure may occur across various time periods over the lifetime. Thus, there may be a critical exposure period that results in particularly large health effects of PM, which is an important consideration for future studies.

Despite these limitations, to the best of our knowledge, this is the first study to evaluate the associations of each level of exposure to ambient PM_10–2.5_ and PM_2.5_ with mean changes in an individual’s laboratory cardiometabolic risks, including fasting glucose and lipid profiles, at 2-year intervals using nationwide cohort data obtained from a large representative Korean population. Few studies have evaluated the effects of size-specific PM on changes in laboratory values, as well as their mechanisms and management. Thus, more studies are needed to identify or prevent this avoidable risk factor.

## Conclusions

In conclusion, this study verifies that higher levels of ambient fine PM exposure were associated with elevated FBG and LDL-C levels in Korean adults. Our findings can help to explain the increased risk of cardiovascular disease and diabetes in areas with higher levels of air pollution and the stronger adverse effects of fine PM on human health than coarse PM. This study highlights the importance of consideration of the size-specific PM-related health effects and public policies targeted at vulnerable groups, including older or physically inactive individuals, to reduce PM-associated harmful effects.

## Data Availability

The data that support the findings of this study are available from the NHIS-NSC but restrictions apply to the availability of these data due to ethical concerns, which were used under license for the current study, and so are not publicly available.

## Abbreviations

PM: Particulate matter
NHIS-NSC: National Health Insurance Service–National Sample Cohort
PM_10_: Particulate matter with aerodynamic diameter ≤ 10 μm
PM_2.5_: Particulate matter with aerodynamic diameter ≤ 2.5 μm
PM_10–2.5_: Coarse particulate matter
FBG: Fasting blood glucose
BMI: Body mass index
CCI: Charlson comorbidity index
95% CI: 95% confidence interval
TC: Total cholesterol
TG: Triglycerides
HDL-C: High-density lipoprotein cholesterol
LDL-C: Low-density lipoprotein cholesterol
